# Mutation analysis of key genes in RAS/RAF and PI3K/PTEN pathways in Chinese patients with hepatocellular carcinoma

**DOI:** 10.3892/ol.2014.2253

**Published:** 2014-06-13

**Authors:** WENMIN HOU, JIBIN LIU, PEIZHAN CHEN, HUI WANG, BANG-CE YE, FULIN QIANG

**Affiliations:** 1 Laboratory of Biosystems and Microanalysis, State Key Laboratory of Bioreactor Engineering, East China University of Science and Technology, Shanghai 200237, P.R. China; 2 Key Laboratory of Food Safety Research, Institute for Nutritional Sciences, Shanghai Institutes for Biological Sciences, Chinese Academy of Sciences, University of Chinese Academy of Sciences, Shanghai 200031, P.R. China; 3 Tumor Institute, Nantong Tumor Hospital, Nantong, Jiangsu 226000, P.R. China

**Keywords:** hepatocellular carcinoma, mutation analysis, *KRAS*, cancer-related genes

## Abstract

The RAS/RAF and PI3K/PTEN signaling pathways play central roles in hepatocarcinogenesis. *KRAS*, *NRAS*, *HRAS*, *BRAF*, *PIK3CA*, *PIK3R1* and *PTEN* are key cancer-related genes in the RAS/RAF and PI3K/PTEN signaling pathways. Genetic alterations in these genes often lead to the dysregulation of the two cascades. Little is known regarding the frequency of hotspot mutations in these critical components among Chinese patients with hepatocellular carcinoma (HCC). In the current study, 57 somatic hotspot mutations in 36 HCCs samples collected from Chinese patients using direct DNA sequencing method were examined. Two cases of *KRAS* somatic mutations (*KRAS* codon 61; Gln to His) were identified among 36 HCCs (5.6%). However, no mutations were found in the *NRAS*, *HRAS*, *BRAF*, *PIK3CA*, *PIK3R1* and *PTEN* genes. These findings indicated that point mutations in the *KRAS* gene, but not mutations in *NRAS*, *HRAS*, *BRAF*, *PIK3CA*, *PIK3R1* and *PTEN* genes, at a somatic level contribute to the abnormal activation of the RAS/RAF and PI3K/PTEN pathways in HCC.

## Introduction

Hepatocellular carcinoma (HCC) is one of the most common malignancies worldwide, accounting for >740,000 new cases and 690,000 mortalities per year (1). Half of these new cases and mortalities were estimated to occur in China. The high rates of HCC in China are largely due to the prevalence of chronic hepatitis B virus (HBV) infection (2). The RAS/RAF and PI3K/PTEN signaling pathways play central roles in hepatocarcinogenesis (3). The aberrant activation of the RAS/RAF and PI3K/PTEN signaling pathways is associated with poor prognosis in cancer patients (4,5). HBV also utilizes the pathways for the control of hepatocyte survival and viral replication (6,7). Mutations of key components (such as *RAS*, *RAF*, *PIK3CA*, *PIK3R1* and *PTEN*) in the RAS/RAF and PI3K/PTEN pathways lead to the dysregulation of the two cascades (8). The *RAS* family comprises three members: *KRAS*, *NRAS* and *HRAS*. Somatic mutations in the *RAS* family are common in numerous human cancer types, including pancreatic, thyroid, colorectal, liver, kidney and lung (9). *BRAF* is the most frequently mutated gene in the *RAF* family, and the *BRAF* mutation has been reported in 61% of melanoma, 53% of papillary thyroid cancer and 11.5% of colorectal cancer patients (10–12). The PI3K gene comprises *PIK3CA,* which encodes the catalytically active p110α subunit, and *PIK3R1,* encoding the p85α regulatory subunit (13). *PIK3CA* is mutated in numerous tumor types, with the frequency ranging from 4 to 32% in breast, colorectal, endometrial, brain, gastric and lung cancer (14–17). *PIK3R1* mutations were identified in 43% of endometrial cancer, 4% of ovarian cancer and 2% of colon cancer (18–19). PTEN acts as a negative regulator of the PI3K pathway and *PTEN* mutations lead to a reduction of its phosphatase activity (20). Mutations of the *PTEN* gene are associated with a wide variety of human tumors (21).

Inhibitors targeting the RAS/RAF and PI3K/PTEN pathways have been developed and the clinical responses of patients were observed to differ according to the genetic alterations of the critical components of the two cascades (22). However, few data are available regarding the prevalence of *KRAS*, *NRAS*, *HRAS*, *BRAF*, *PIK3CA*, *PIK3R1* and *PTEN* mutations in Chinese patients with HCC. In the present study, we conducted mutational analysis of 57 somatic hotspot mutations in *KRAS*, *NRAS*, *HRAS*, *BRAF*, *PIK3CA*, *PIK3R1* and *PTEN* in 36 Chinese patients with HCC.

## Materials and methods

### Patients and tissue samples

Thirty-six patients with HCC undergoing surgery at Nantong Tumor Hospital (Nantong, China) between 2009 and 2011 were enrolled in this study. Tumor samples and adjacent normal liver tissues from the corresponding patients were fixed with 10% formalin, embedded in paraffin and stained with hematoxylin and eosin (H&E). Tumor staging was performed according to the Barcelona Clinic Liver Cancer (BCLC) staging classification (23). This study was approved by the Ethics Committee of Nantong Tumor Hospital. Written informed consent was obtained from each patient prior to sample collection.

### Genomic DNA extraction

Tumor areas and non-tumorous tissue areas were identified on H&E-stained slides. Genomic DNA was extracted from formalin-fixed paraffin-embedded tissues of HCC with the QIAamp DNA FFPE Tissue kit (Qiagen GmbH, Hilden, Germany) according to the manufacturer’s instructions. Briefly, samples were placed into Eppendorf tubes and the paraffin was removed. Next, the tubes were incubated with proteinase K (Qiagen GmbH) at 56°C for 1 h. Following proteinase K digestion, the samples were incubated at 90°C for 1 h and DNA was extracted using QIAamp MinElute columns (Qiagen GmbH).

### Mutation analysis

Polymerase chain reaction (PCR) was performed to amplify the gene fragments including the hotspot mutations shown in Table I. The selection of the hotspots was based on the prevalence of mutations in cancers identified in the COSMIC database (24). A 50 μl volume of PCR was prepared using the Taq PCR Master Mix kit (Qiagen GmbH), according to the manufacturer’s instructions. The thermocycling was performed at 94°C for 3 min; 35 cycles of 94°C for 30 sec, 56°C for 30 sec and 72°C for 60 sec; followed by a final 10 min at 72°C. PCR products were run on 1.5% agarose gel electrophoresis and visualized with ultraviolet light to confirm sizes. DNA purification was performed with the QIAprep Gel Extraction kit (Qiagen GmbH) according to the manufacturer’s instructions. Briefly, the DNA fragments were excised from the agarose gel with a scalpel and placed in a colorless tube. DNA cleanup was conducted using QIAquick spin columns (Qiagen GmbH). Direct DNA sequencing was performed using a Big Dye Terminator (v3.1) kit (Applied Biosystems, Foster City, CA, USA). The sequencing products were run on an Applied Biosystems 3130XL Genetic Analyzer (Applied Biosystems). DNA sequencing results were analyzed using Chromas software (Technelysium, Brisbane, Queensland, Australia). The primers used for the PCR are listed in Table I.

## Results

### Clinicopathological characteristics

Of 36 patients with HCC, the median age was 54 years (range, 40–77 years), including 33 males and three females. The majority of the cases had HCC associated with HBV infection (34/36; 94.4%). All patients were negative in hepatitis C virus infection. The concentrations of serum AFP of 16 patients (16/36; 44.4%) were higher than 400 ng/ml. The BCLC staging classification was used to classify the cancer staging (23). There were 2, 25, 8, 1 and 0 cases of stages 0 to D, respectively (Table II).

### Mutation analysis of key genes in the RAS/RAF and PI3K/PTEN pathways

**Figure 1:**
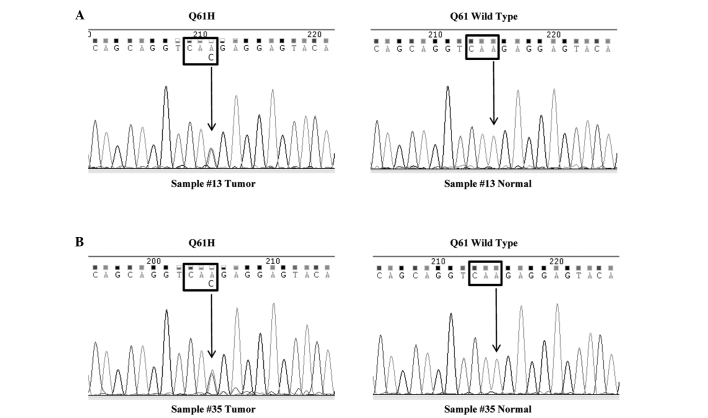
Sequence analysis of *KRAS* gene mutation in codon 61 in two hepatocellular carcinoma cases. Sequence chromatograms of codon 61 in tumor and normal tissues from (A) Sample #13 and (B) Sample #35.

We analyzed hotspot-containing gene fragments of key genes in RAS/RAF and PI3K/PTEN pathways using PCR amplification followed by direct sequencing. The hotspots were listed in Table I. In all, two samples (Sample #13 and #35) had point mutations in codon 61 (Q61H) of the *KRAS* gene and the mutation rate was 5.6% (Fig. 1). In the two cases, codon 61 was altered from CAA, coding for Gln, to CAC, coding for His. To confirm the two mutations occurred at the somatic level, we tested codon 61 mutation status in non-tumorous tissues from the two patients. The results showed that codon 61 was wild-type in normal tissues from Sample #13 and #35 (Fig. 1). The two patients harboring *KRAS* mutation were male. Patient no. 13 was 49 years old, had HBV infection and stage A HCC, and an AFP level of 3.4 ng/ml. Patient no. 35 was 69 years old, had stage B HCC and was negative for HBV infection, with an AFP level of 1.95 ng/ml. No other mutations in the *HRAS*, *NRAS*, *BRAF*, *PIK3CA*, *PIK3R1* and *PTEN* genes were identified.

## Discussion

Targeting the RAS/RAF and PI3K/PTEN pathways are novel therapeutic strategies that may be exploited for the treatment of HCC (8). As the RAF-kinase inhibitor sorafenib has been demonstrated to be effective in the treatment of HCC, *BRAF* mutations have become a favored target in HCC treatment recently (25). However, the somatic mutation prevalence and distribution of the key genes in the two pathways remain largely unknown in Chinese patients with HCC. Therefore, the present study set out to examine the frequency of hotspot mutations of the *KRAS*, *NRAS*, *HRAS*, *BRAF*, *PIK3CA*, *PIK3R1* and *PTEN* genes in 36 human HCC tissues from Chinese patients. Only *KRAS* somatic mutations were identified, with a mutation rate of 5.6%.

The incidence of *KRAS* mutations has been found in 80% of advanced pancreatic cancer (26), 45% of cholangiocarcinoma (27) and 32% of colorectal cancer (28) patients. COSMIC database has shown that mutations in codons 12, 13 and 61 of the *KRAS* gene are known hotspots in various types of cancer. The frequency and distribution of *KRAS* mutation in HCC from several previous studies are summarized in Table III. The majority of these studies have shown that *KRAS* gene mutations occur infrequently (<10%) in HCC. The codon 12 accounts for the majority of *KRAS* mutations detected (~70%), whereas mutations affecting codon 13 and codon 61 account for the remaining 30%. One third of the twelve studies did not evaluate the *KRAS* codon 61 mutation status, which may cause bias in the distribution of the *KRAS* mutation. Three whole exome sequencing studies conducted mutational screening in all *KRAS* exons and found that the mutations were clustered in the hotspots (29–31). In the current study, mutations were detected in codons 12, 13 and 61 of the *KRAS* gene, and two out of 36 (5.6%) HCCs harbored *KRAS* mutations in codon 61. Therefore, *KRAS* gene mutations may participate in hepatocellular carcinogenesis.

The present study also investigated the hotspot mutations in *NRAS* and *HRAS*, but found no mutation in the two genes. Few studies have focused on the mutation incidence of these two *RAS* family members in Chinese patients with HCC. A whole exome sequencing study identified no mutation in these two genes in a Chinese population (31). Challen *et al* found that the frequency of *NRAS* mutations was 15.8%, but did not identify *HRAS* mutations, in British patients with HCC (32). Taketomi *et al* reported neither *NRAS* nor *HRAS* mutations were detected in Japanese HCC cases (33). Thus, the mutational activation of *NRAS* and *HRAS* genes is an uncommon event in the pathogenesis of HCCs.

*BRAF* mutations can abnormally activate downstream signaling pathways in HCC and act as indicator of cetuximab resistance in patients with colon cancer (34,35). *BRAF* mutations are believed to be rare in HCCs. Previously, no *BRAF* mutations were identified in German and Chinese populations (27,36). However, Colombino *et al* detected that the *BRAF* gene was highly mutated in ~23% of Italian HCC cases (37). In the current series, no *BRAF* mutations were observed, indicating that *BRAF* mutation does not play a major role in abnormal activation of RAS/RAF signaling pathway.

*PIK3CA*, *PIK3R1* and *PTEN* are key genes in the PI3K/PTEN pathway (8). In the current study, it was found that mutations were absent in the three genes. Previously, *PIK3CA* was observed to be frequently mutated in Korean and Italian patients with HCC, with mutation rates of 35.6 and 28%, respectively (15,37). However, Tanaka *et al* did not identify *PIK3CA* mutations in Japanese patients with HCC, and Riener *et al* reported that the *PIK3CA* mutation incidence was 2% in Swiss patients with HCC (38,39). In two studies in Chinese patients with HCC, the mutation rates were 1.6 and 1.1% (36,40), which were similar to those of the present study. The conflicting data may be due to the different genetic backgrounds of the populations, HBV infection status and smaller sample size in the current study. *PIK3R1* mutation has been found to occur infrequently in numerous cancer types, including ovarian and colon cancer (19), and the present study showed a low frequency of alteration of *PIK3R1* in HCC. Inactivation of *PTEN* in HCC may be largely due to frequent loss of heterozygosity of the *PTEN* allele; the frequency was identified to be ≤44.4% (41). Wang *et al* investigated *PTEN* mutations in exons 5 and 8, but failed to detect any (42), which was in agreement with the results of the present study. Mutations in the *PIK3CA*, *PIK3R1* and *PTEN* genes rarely occur in HCC, suggesting that somatic point mutations of these three genes may not play an important role in HCC in the Chinese population. However, further research is necessary to confirm these results in larger sample size.

In summary, the present study investigated the prevalence of *KRAS*, *NRAS*, *HRAS*, *BRAF*, *PIK3CA*, *PIK3R1* and *PTEN* mutations in 57 hotspot mutations. Two cases of *KRAS* mutation were identified among 36 HCC cases. The findings indicated that point mutations in the *KRAS* gene, but not mutations in the *NRAS*, *HRAS*, *BRAF*, *PIK3CA*, *PIK3R1* and *PTEN* genes, at the somatic level contribute to the abnormal activation of the RAS/RAF and PI3K/PTEN pathways in HCC. Considering the low frequency of key genes in the RAS/RAF and PI3K/PTEN signaling pathways, other mechanisms to activate the RAS/RAF and PI3K/PTEN pathways, such as gene amplification, deletion, and aberrant methylation, may be involved in the development and progression of HCC.

## Figures and Tables

**Table I:** Primers used for polymerase chain reaction in this study.

Gene	Mutation	Forward primer sequence (5′ to 3′)	Reverse primer sequence (5′ to 3′)
KRAS	G12C, G12D, G13S, G13D, L19F, Q22K	CTTAAGCGTCGATGGAGGAG	CCCTGACATACTCCCAAGGA
	A59T, Q61E, Q61R, Q61H	GGTGCTTAGTGGCCATTTGT	CCTAGGTTTCAATCCCAGCA
	A146T	TTGTGGACAGGTTTTGAAAGA	AGAAACCAAAGCCAAAAGCA
NRAS	G12S, G12V, G13R, G13V, A18T	GCCCAAGGACTGTTGAAAAA	CCGACAAGTGAGAGACAGGA
	Q61K, Q61R, Q61H	GGCAGAAATGGGCTTGAATA	AGGTTAATATCCGCAAATGAC
HRAS	G12S, G12V, G13R, G13D	GTGGGTTTGCCCTTCAGAT	TGGTGGATGTCCTCAAAAGA
	Q61K, Q61R, Q61H	TGGCTGTGTGAACTCCCC	GTCAGTGAGTGCTGCTCCC
BRAF	G464V, G466V, G469A, V471F	CACTTGGTAGACGGGACTCG	AGTTTATTGATGCGAACAGTGA
	D594G, L597V, V600E	AACTCTTCATAATGCTTGCTCTGA	AGCCTCAATTCTTACCATCCA
PIK3CA	R38G, Q75E, R108H	GCCTAATCAAGTCAAACTATGGAA	AAGCTTTATGGTTATTTGCATTTT
	G118D	ATGTTTGCTGCCTTTGCTCT	ATAAGCAGTCCCTGCCTTCA
	C378R	TAAGGGGATTGTGGGCCTAT	AATGGGGTCTTGCTTTGTTG
	C420R	CTCATGCTTGCTTTGGTTCA	TTGGCATGCTCTTCAATCAC
	P539R, E542K, E545K, E545G, Q546K	GATTGGTTCTTTCCTGTCTCTG	CCACAAATATCAATTTACAACCATTG
	T1025A, M1043T, M1043I, H1047Y, H1047R, H1047L	CATTTGCTCCAAACTGACCA	CACCCCAAGCATTTTTCTTC
PIK3R1	G376R	CAGACGGGACCTTTTTGGTA	AACAAAATAGCTGACATGGAAACA
	K459E, D464H	GGCTTCTCTGACCCATTAACC	CCCCACCTCATTCGTAAAAA
	L570P	GGAAGAGAAGCCACGCTTTA	CCCAACCACTCGTTCAACTT
PTEN	R130G, R130Q	CCGTATAGCGTAAATTCCCAGA	TCTCAGATCCAGGAAGAGGAA
R233X	TGCTTGAGATCAAGATTGCAG	GCCATAAGGCCTTTTCCTTC	

**Table II:** Clinical characteristics of hepatocellular carcinoma patients.

Characteristic	Value
Age, years	
Median (range)	54 (40–77)
Gender, n (%)	
Male	33 (91.7)
Female	3 (8.3)
Etiology, n (%)	
HBV(+)	34 (94.4)
HBV(−)	2 (5.6)
AFP, n (%)	
>400 ng/ml	16 (44.4)
≤400 ng/ml	20 (55.6)
Stage, n (%)	
0	2 (5.6)
A	25 (69.4)
B	8 (22.2)
C	1 (2.8)
D	0 (0.0)

**Table III:** Reported point mutations in codons 12, 13, and 61 of *KRAS* in hepatocellular carcinomas.

Author (ref)	Population	No. of patients	Codon 12	Codon 13	Codon 61	Frequency (%)
Zuo *et al* (36)	Chinese	64	2	1	NA	4.7
Huang *et al* (31)	Chinese	10	0	0	0	0.0
Taketomi *et al* (33)	Japanese	61	0	1	0	1.6
Tsuda *et al* (43)	Japanese	30	1	0	0	3.3
Taniguchi *et al* (44)	Japanese	15	0	0	NA	0.0
Fujimoto *et al* (29)	Japanese	27	0	0	0	0.0
Tada *et al* (45)	Japanese	12	0	0	0	0.0
Bose *et al* (46)	Indian	30	2	0	0	6.7
Tannapfel *et al* (27)	German	25	0	0	NA	0.0
Weihrauch *et al* (47)	German	20	3	0	NA	15.0
Challen *et al* (32)	British	19	0	NA	1	5.3
Guichard *et al* (30)	French	149	1	0	1	1.3
Colombino *et al* (37)	Italian	65	1	0	0	1.5

NA, not available.
